# CpG Methylation Changes within the *IL2RA* Promoter in Type 1 Diabetes of Childhood Onset

**DOI:** 10.1371/journal.pone.0068093

**Published:** 2013-07-12

**Authors:** Marie-Pierre Belot, Delphine Fradin, Nga Mai, Sophie Le Fur, Diana Zélénika, Julie Kerr-Conte, François Pattou, Bruno Lucas, Pierre Bougnères

**Affiliations:** 1 INSERM U986 and Department of Pediatric Endocrinology, Bicêtre Hospital, Pôle I3E, Paris Sud University, France; 2 Centre National de Génotypage, Evry, France; 3 INSERM U859, Université Lille Nord de France and Centre Hospitalier Régional Universitaire de Lille, Lille, France; 4 CNRS Unité Mixte de Recherche 8104, INSERM U1016, Cochin Hospital, Paris, France; University of Michigan Medical School, United States of America

## Abstract

None of the polymorphic variants of the *IL2RA* gene found associated with Type 1 Diabetes (T1D) was shown to have a functional effect. To test if the epigenetic variation could play a role at this locus, we studied the methylation of 6 CpGs located within the proximal promoter of *IL2RA* gene in 252 T1D patients compared with 286 age-matched controls. We found that DNA methylation at CpGs −373 and −456 was slightly but significantly higher in patients than in controls (40.4±4.6 vs 38.3±5.4, p = 1.4E4; 91.4±2.8 vs 89.5±5.3, p = 1.8E-6), while other CpG showed a strictly comparable methylation. Among 106 single nucleotide polymorphisms (SNPs) located in the neighboring 180kb region, we found that 28 SNPs were associated with DNA methylation at CpG −373. Sixteen of these SNPs were known to be associated with T1D. Our findings suggest that the effect of IL2RA risk alleles on T1D may be partially mediated through epigenetic changes.

## Introduction

Type 1 Diabetes (T1D) is characterized by an autoimmune destruction of pancreatic β cells, a process in which autoreactive T cells play a pivotal role [1]–[3]. IL2RA (IL-2 receptor α-chain, CD25) is part of the high-affinity IL-2 receptor complex. *IL2RA* is expressed constitutively on regulatory T cells, a population of T cells that have a potent ability to suppress autoreactive T cells [4], whereas is induced in other T cells. *IL-2RA* polymorphisms are associated with T1D [5]–[8] and other autoimmune diseases such as multiple sclerosis or rheumatoid arthritis [9], [10].

Six positive regulatory region (PRR) and two negative regulatory elements (NRE) located between −9 kb and +3.6 kb around the transcriptional start site (TSS) are implicated in the regulation of *IL2RA* expression in response to stimuli [11]. No disease-associated SNPs have been reported in these regions. However, each of these regions encompasses several CpGs known to modify gene expression by altering the binding of transcriptional proteins or by allowing the binding of methyl-CpG binding domain proteins. DNA methylation changes have also been shown to be important for the selective transcription of cytokine genes in T cell subsets [12], [13].

For these reasons, we studied the DNA methylation status of 6 CpGs located in the proximal promoter of *IL2RA* in T1D patients together with the genetic variants located on the surrounding 180 kb region of chromosome 10p15.1.

## Results

### DNA Methylation Specific Pattern across Tissues

**Figure 1:**
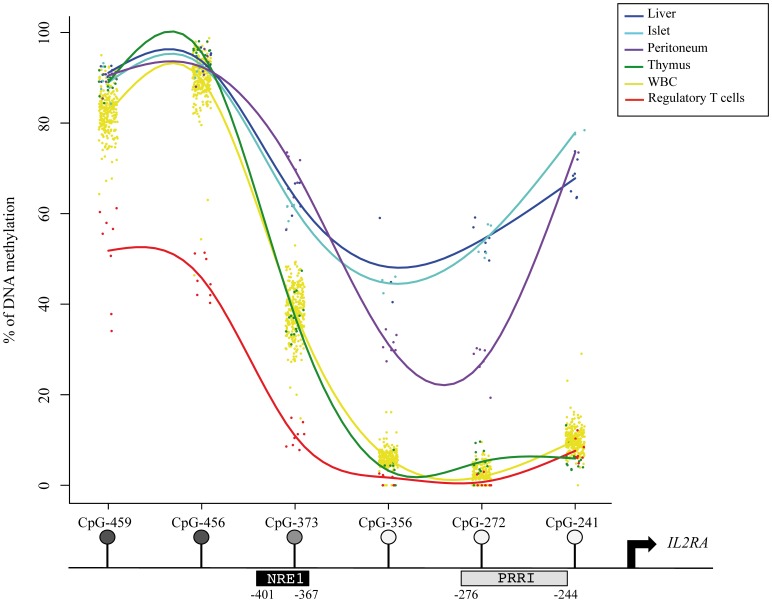
Schematic representation of DNA methylation levels in the proximal promoter of the *IL2RA* gene. Tissues come from different non-diabetic controls: WBC (n = 286), liver (n = 7), peritoneum (n = 8), thymus (n = 16) and Langerhans islets (n = 7), regulatory T cells (n = 8).

The pattern of methylation in the whole blood cells (WBC) of 286 non-diabetic individuals (Table 1) showed important variations across the 6 studied CpGs located in the proximal promoter region of the *IL2RA* gene (Figure 1). CpGs −241, −272 and −356, close to the TSS, are almost unmethylated whereas the more distant CpGs −456 and −459 are almost completely methylated and CpG −373 had an intermediate level of methylation. This global pattern of methylation was also seen in T1D patients, with subtle changes that will be discussed. Methylation of the studied CpG was different in other tissues. CpGs −241, −272 and −356 showed an intermediate DNA methylation level in liver, islet and peritoneum and remained unmethylated in the thymus. CpG −373 showed also a higher methylation level in liver, islet or peritoneum than in WBC and thymus. Methylation of CpGs −456 and −459 was comparable across studied tissues. The level of methylation at CpGs −459, −456 and −373 was lower in regulatory T cells.

**Table 1:** Main characteristics and CpG methylation levels in the *IL2RA* promoter of T1D patients and age-matched non-diabetic controls.

	T1D Patients	Controls	pvalue
**N**	252	286	
**Sex (M/F)**	132/120	130/156	
**Current age (yrs)**	11.45±4.13	11.08±3.09	0.19
**BMI (kg/m^2^)**	18.67±3.07	21.63±9.51	0.86
**Age at clinical onset (yrs)**	6.22±3	–	–
**Diabetes Duration**	5.24±3.71	–	–
**Hba1c**	8.09±1.32	–	–
**CpG-241**	9.74±1.91	9.94±2.72	0.71
**CpG-272**	1.7±1.79	1.92±2.02	0.38
**CpG-356**	6.24±1.27	5.94±2.11	0.017
**CpG-373**	40.38±4.64	38.34±5.46	1.4.10^−4^
**CpG-456**	91.43±2.77	89.5±5.3	1.79.10^−6^
**CpG-459**	82.32±4.5	82.34±4.57	0.82

Results are expressed as mean ± sd.

### Differential DNA Methylation is Related to T1D Status

**Figure 2:**
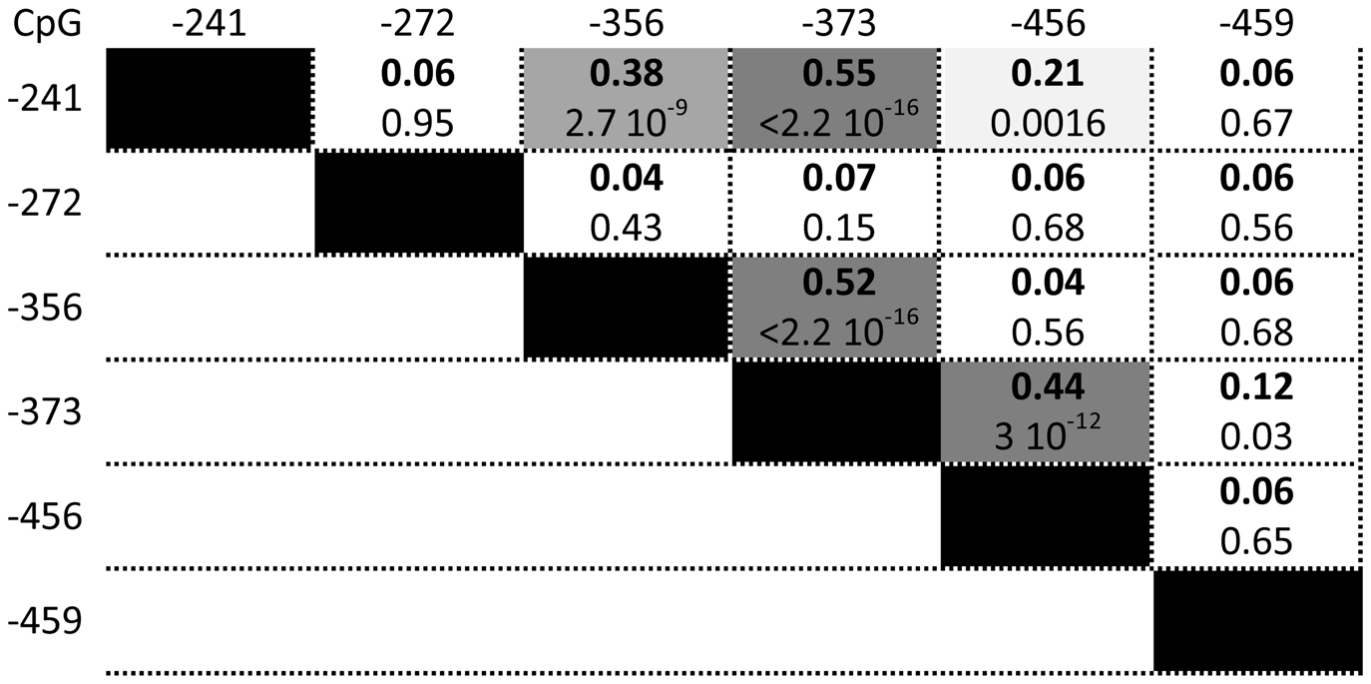
Correlation matrix of the methylation values (%) at the *IL2RA* promoter CpG sites in T1D patients (R in bold, p-value below).

The comparison of the 252 T1D patients with 286 age-matched controls (Table 1) showed no T1D-related global directional change in DNA methylation level that would affect all CpGs equally (Table 1). There were, however, significant differences at the specific CpG level. T1D patients had a higher level of CpGs −373 and −456 methylation than controls (p = 1.10^−4^ and p = 2.10^−6^ respectively). Methylation levels of these two CpGs were closely correlated (r = 0.44, p<3.10^−12^, Figure 2), suggesting a shared regulation of the methylation status of these CpG residues. CpG−356 showed a slight and less significant increase in percent of methylation in T1D group (p = 0.02), while methylation of the three other CpGs was comparable with controls (Table 1).

We found no relationship between CpG methylation levels at any position with age at diagnostic, current glycemic status reflected by glycated haemoglobin (HbA1c) or T1D duration, except for CpG −241 that showed a slight increase of methylation with diabetes duration (p = 0.004, Figure S1).

### Influence of SNP Genotypes upon CG Methylation at the IL2RA Locus

Among the analyzed 106 SNPs located within 180kb of the chromosome 10p15.1, we found 32 SNPs that were associated with DNA methylation of CpG −241, −272, −356 and −373 (Table 2). Twenty of these 32 SNPs were previously shown to be associated with T1D by GWAS [5], [14], [15].

**Table 2:** SNPs associated with DNA methylation at *IL2RA* promoter locus.

SNP	position (hg18)	p-value	FDR	Associated CpG	GWAS p-value*
**rs942201**	chr10∶6,126,298	0.0008	0.05	−272	1.1E-7
**rs10905669**	chr10∶6,132,099	1.4E-03	4.9E-02	−272	5.8E-9
**rs8177775**	chr10∶6,052,396	6.2E-04	2.0E-02	−241	0.05
**rs8177772**	chr10∶6,055,063	6.2E-04	2.0E-02	−241	0.05
**rs6602360**	chr10∶6,069,732	0.006	0.02	−373	1.2E-4
		0.002	0.02	−241	
**rs7911500**	chr10∶6,077,732	0.002	0.01	−373	
**rs7898880**	chr10∶6,077,559	0.001	0.008	−373	1.0E-4
**rs10795737**	chr10∶6,089,350	3.5E-05	0.0007	−373	
**rs12722588**	chr10∶6,100,439	0.003	0.01	−373	
**rs7900744**	chr10∶6,105,617	0.002	0.009	−373	4.8E-3
**rs2025345**	chr10∶6,107,694	3.4E-05	0.0007	−373	0.01
		0.0004	0.02	−241	
**rs12722561**	chr10∶6,109,899	0.0001	0.002	−373	
		4.6E-04	2.4E-02	−356	
		0.002	0.02	−241	
**rs7910961**	chr10∶6,117,802	0.001	0.007	−373	0.02
**rs12722523**	chr10∶6,118,396	0.0003	0.003	−373	
		0.002	0.02	−241	
**rs7100984**	chr10∶6,118,545	0.0007	0.004	−373	
**rs7072398**	chr10∶6,119,852	0.0005	0.004	−373	
**rs12722515**	chr10∶6,121,236	0.0003	0.003	−373	
		0.002	0.02	−241	
**rs4749924**	chr10∶6,122,402	0.002	0.008	−373	5.8E-4
**rs6602398**	chr10∶6,122,959	5.9E-07	6.3E-05	−373	9.0E-5
		7.5E-04	0.02	−241	
**rs4749926**	chr10∶6,125,318	1.2E-06	6.3E-05	−373	6.6E-3
		1.3E-03	4.9E-02	−272	
		1.0E-03	0.02	−241	
**rs791587**	chr10∶6,128,705	4.7E-06	0.0002	−373	
		0.005	0.04	−241	
**rs791589**	chr10∶6,129,577	0.002	0.009	−373	0.03
		0.001	0.02	−241	
**rs2104286**	chr10∶6,139,051	0.0002	0.003	−373	2.9E-9
		1.2E-03	0.02	−241	
**rs7090512**	chr10∶6,150,835	0.0001	0.002	−373	1.9E-4
**rs4749955**	chr10∶6,158,672	0.0008	0.005	−373	1.4E-3
**rs11594656**	chr10∶6,162,015	0.0007	0.004	−373	2.7E-5
**rs6602437**	chr10∶6,170,083	0.0003	0.003	−373	2.6E-3
		1.3E-03	0.05	−356	
**rs10905806**	chr10∶6,192,316	0.001	0.008	−373	0.03
**rs6602450**	chr10∶6,193,303	0.0003	0.003	−373	
**rs4749997**	chr10∶6,201,787	0.0004	0.003	−373	
**rs7905816**	chr10∶6,209,278	0.002	0.01	−373	
**rs4750005**	chr10∶6,209,691	0.002	0.009	−373	4.1E-3
		2.3E-04	2.4E-02	−356	

* p-value from Todd et al. 2007, Barrett et al. 2009 and Bradfield et al. 2011.

**Figure 3:**
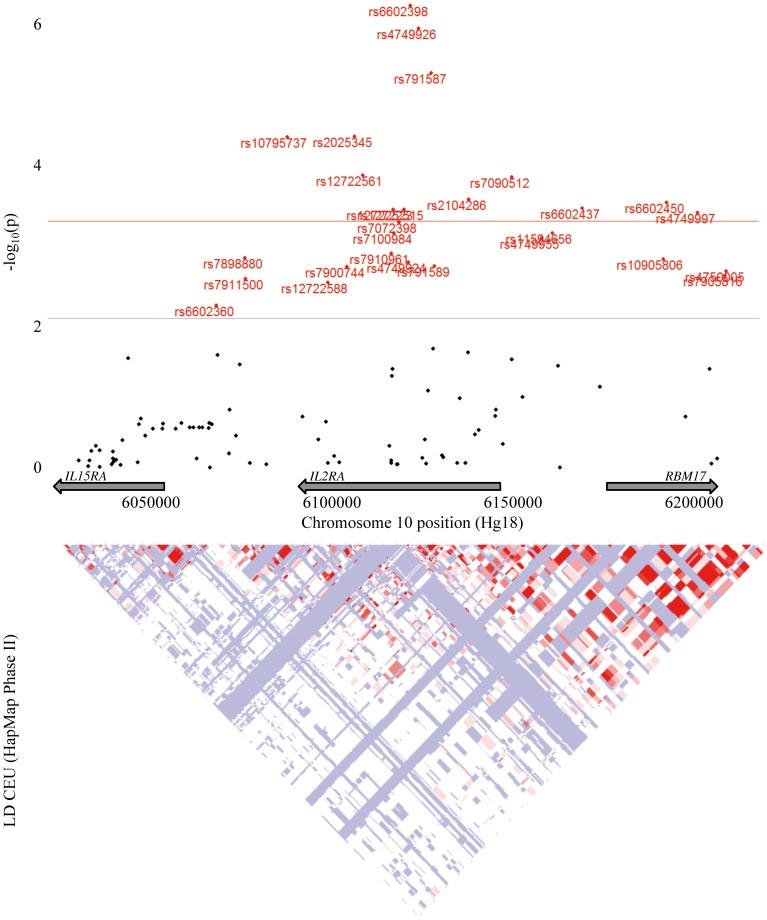
Association results for the *IL2RA* region and CpG −373. Manhattan plot represented p-values of Wald test based on linear regression model (for each SNP, association test performed in plink which compares the quantitative phenotype means for three genotypes). The red horizontal line represents the significance threshold of P = 4.7×10^−4^.

The association between rs6602398 and CpG -373 was the strongest observed, with methylation of 42%, 40%, and 37% in the GG, GT, and TT genotypes respectively. DNA methylation level at CpG −373 showed an association with 28/32 SNPs located from 23 kb to 116 kb of this CpG, and 16/28 SNPs previously found to be associated with T1D (Figure 3).

We observed no genetic-epigenetic statistical correlation between SNPs and DNA methylation at CpGs −456 and −459.

## Discussion

Using a candidate locus approach, the current study of CpG methylation in the *IL2RA* promoter found that differences could be detected in T1D patients. Two CpGs (−373 and −456) showed an increased methylation in T1D patients compared to controls. These significant differences were of small magnitude, but were in the range previously reported for CpG in other gene promoters in case control studies [16], [17]. These methylation differences could also be described in a categorical manner to simplify the presentation of results: if classified as low (<1 Standard Deviation (SD)), intermediate and high (>1SD), the methylation of CpGs −373 and 456 indicates that 4.6% of T1D patients *versus* 2.6% of controls were in the upper category.

It was interesting to see that DNA methylation at CpG −373 was correlated with 16 SNPs located at the *IL2RA* locus and previously known to be associated with T1D [5], [14], [15].

The most significant association with CpG −373 methylation was seen for rs6602398 and rs4749926 (6.3E-05) that are both located on intron 1. The *IL2RA* region encompasses large blocks of linkage disequilibrium and it is possible that part of the genetic-epigenetic associations observed is supported predominantly by only a few tag SNPs. These associations allow to postulate the existence of short distance genetic-epigenetic interactions resembling the allele-specific methylation phenomenon reported at the *INS* [16] or *FTO* locus [18]. Our observation is consistent with the fact that loci harboring genetic variants that influence methylation state, called methylation quantitative trait loci (methQTL) [19]–[22], are known to be preferentially located in *cis,* outside CpG islands and within a variable distance from CpG sites that averages about 81 Kb [19].

Among the SNPs associated with CpG−373 methylation was the rs2104286 variant (p = 2.9E-9), located in the 5′end of the long intron 1, that is the third independent IL2RA marker of T1D [23]. The odds ratio for T1D is 1.57 (CI 1.25–1.99) for AA homozygotes at this SNP [23]. In our T1D cohort, rs2104286 AA carriers showed a 4% decrease in DNA methylation level in CpG −373 compared to GG carriers, with an intermediate level for heterozygotes.

The two other SNPs that tag the first and the second T1D-associated haplotypes are rs12722495 (not available in our genotyping arrays) and rs11594656 that shows a strong association with the methylation of CpG −373 (p = 2.7E-5). Homozygote risk allele carriers are more methylated at CpG −373 than other homozygotes or heterozygotes. The finding of a link between genetic variants and epigenomic marks in T1D patients at the *IL2RA* locus may provide a more general example of how genetic and epigenetic variation can be related. This may help our future understanding of the “missing heritability” enigma seen with many genome wide association studies of multifactorial traits or diseases, if it proves true that SNPs are only the markers that tag neighboring epigenomic variations. According to this view, certain SNPs showing strong statistical association with complex diseases may not have any functional effects *per se*, but may be associated with epitypes that have functional effects on gene expression. It could be the case of rs11594656, where carriers of risk alleles are more methylated and leading probably to less expression of *IL2RA* gene.

According to our results in various tissues, the *IL2RA* promoter region could be defined as a T-DMR (tissue-specific differentially methylated region). T-DMRs are known to be involved in the expression of tissue-specific genes as well as of key transcription factors that govern transcription networks and tissue specificity [24]–[30]. Peripheral blood being the only readily accessible tissue sample for epidemiological studies, initial reports indicated that DNA methylation measured in WBC may in some instances be informative when tissues where the disease originates are not available [31]–[33]. In this respect, most interesting cells to study the methylation of the IL2RA gene promoter are regulatory T cells in which IL-2 signaling seems to be a major effector in the pathophysiology of T1D [34]. However, since T cells represent 40–60% of WBC and regulatory T cells expressing IL2RA only 10% of peripheral CD4+ and <1% of CD8+ [35], this was beyond the reach of our study. To get *IL2RA* expressing cells, large amounts of WBC can be freshly sorted, but this needs exceedingly large blood sample not accessible in clinical research in children or adolescents and was not feasible from our preexisting DNA bank. This is why, we confined our study to a subset of 8 healthy persons, in whom we found that the level of DNA methylation in the IL2RA promoter of regulatory T cells was lower than in other WBC while being correlated with methylation in other blood cells (figure S2). Whether this has a functional meaning cannot be known from the current data. IL2RA is also expressed in a variety of hematopoietic cell types, including activated T and B-lymphocytes [36], [37], NK (Natural Killer) cells [38]–[40], monocytes [41], [42] and a subset of dendritic cells [43], [44].

In conclusion, the picture of genetic and epigenetic variation at a T1D risk locus was shown by the current study to be both entangled and complex. A given risk allele of a T1D associated SNP is associated with increased methylation at a risk CpG, while another risk allele of another T1D-associated SNP is associated with decreased methylation. The overall level of methylation of the risk CpG being increased in T1D patients, it is clear that the genetic influence of the studied SNPs was not the sole factor that could explain the changes in methylation observed in the T1D patients. Only 1.7% of the variance of methylation at CpG −373 could be attributed to rs2104286 and 4.6% to rs11594656. This leaves a large contribution to influences from other genetic variants or environmental factors shaping the methylation of CpG throughout development. We do not think that the observed methylation changes in T1D could be attributed to the disease status, since they were independent from diabetes duration or HbA1c, although subtle T1D-associated environmental factors acting for example through dietary changes could be important in determining DNA methylation level in specific positions (il faudrait une ref indiquant un exemple de cette affirmation).

## Materials and Methods

### Participants

Whole blood cell samples were obtained from 252 patients and 286 controls were randomly extracted from the ISIS-Diab cohort [16]. Patients and controls were included in the study according to the French bioethics law. Families were carefully informed and signed a detailed informed consent. Ethical approval for the study was given by the Ethical Review Board of Ile de France (DC-2008-693 NI 2620, CPP) and CNIL (DK-2010-0035).

### Tissues

Samples from human liver (n = 7), peritoneum (n = 8), thymus (n = 16) and Langerhans islets (n = 7), were provided by F. Pattou and J. Kerr-Conte (UMR1011, U859, Lille). Each tissue comes from a different person.

### Isolation of Regulatory T cells

Human CD4+CD25+ T cells were isolated from 50 ml of peripheral blood of 8 randomly chosen healthy blood donor volunteers using density gradient centrifugation (Lymphocytes separation medium, Eurobio) and purified by Human CD4+CD25+ regulatory T Cell Isolation Kit according to manufacturer’s instructions (Miltenyi). Briefly, CD4+ T cells were negatively selected from the total PBMCs. Positive selection with anti-CD25 magnetic microbeads was then used to separate the negative CD4+CD25− T cell fraction from the CD4+CD25+ T cells. Cells were applied to a second magnetic column, washed and eluted again. This procedure led to the complete positive selection of CD4+CD25+ T cells. Genomic DNA was extracted using Gentra Puregene DNA isolation kit (Qiagen).

### Isolation of Genomic DNA and Bisulfite Genomic Conversion

Nucleic acids were extracted from whole blood cells (WBC) or tissue samples using the phenol chloroform method. Genomic DNA was treated with EZ-96 DNA Methylation-Gold Kit, according to manufacturer’s protocol (Zymo Research Corporation).

### Pyrosequencing

We PCR-amplified the bisulfite treated genomic DNA using unbiased primers (sequences on request) and performed quantitative pyrosequencing. Pyrosequencing was performed using a PyroMark Q96 ID Pyrosequencing instrument (Qiagen). Pyrosequencing assays were designed using MethPrimer (http://www.urogene.org/cgi-bin/methprimer/methprimer.cgi). Briefly, 200 ng of genomic DNA was treated with EZ DNA Methylation-Gold Kit and amplified. Biotin-labeled single stranded amplicons were isolated according to protocol using the Qiagen Pyromark Q96 Work Station and underwent pyrosequencing with 0.5µM primer. The percent methylation for each of the CpGs within the target sequence was calculated using PyroQ CpG Software (Qiagen).

### Genotyping and Quality Control

Genotyping was attempted for 252 T1D samples on the Illumina HumanHap300 array and the Illumina Human610-Quad BeadChips at the Centre National de Génotypage (CNG, France). Standard quality control procedures were applied. Briefly, genotype data were retained in the study for samples that had been successfully genotyped for >95% of the SNP markers. SNPs with call rates of <98%, with MAF <2% or showing departure from Hardy-Weinberg equilibrium in the control population (P<10^−3^) were excluded. For our study, we selected polymorphisms located on chromosome 10, from 6,030,000 to 6,210,000 bp (Hg18), corresponding to 106 SNPs.

### Statistical Analysis

Differences in DNA methylation of the IL2RA promoter between T1D and non-diabetic controls were analyzed using non-parametric Wilcoxon rank sum test. Correlations were calculated as adjusted R square that measures the proportion of the variation in the independent variable accounted for by the explanatory variables. The analysis of differences in DNA methylation of the IL2RA promoter according to genotypes was performed in PLINK. For each SNP, we compared the methylation mean for the three genotypic states using the Wald test statistic, based on a linear regression model, to generate a p-value. Associations were considered significant when p-value<0.05/106 = 4.7E-04. The results were visualized as a Manhattan plot. Methylation analyses adjusted for age were conducted using logistic regression. Results are expressed as mean ± sd. All statistical analysis were conducted using R2.14.2.

## Supporting Information

Figure S1 **Lack of correlation between age at diagnostic, Hba1c and diabetes duration and** ***IL2RA*** **promoter methylation in T1D patients.** Only CpG −241 showed a slight trend with diabetes duration (p = 0.004).
(TIF)

Figure S2 **Correlation between** ***IL2RA*** **promoter methylation in regulatory T cells and other blood cells from 8 healthy individuals.** Only CpG −356 showed a significant correlation however, all other CpG seemed correlated but failed to reach the significativity because of the weak number of participants.
(TIF)

Table S1 **List of the 77 diabetic centers by alphabetic order participating to the ISIS-DIAB network.**
(DOCX)

## References

[1] Castano L, Eisenbarth GS (1990) Type-I diabetes: a chronic autoimmune disease of human, mouse, and rat. Annu Rev Immunol 8: 647–679. 2188676 10.1146/annurev.iy.08.040190.003243

[2] Roep BO (2003) The role of T-cells in the pathogenesis of Type 1 diabetes: from cause to cure. Diabetologia 46: 305–321. 12687328 10.1007/s00125-003-1089-5

[3] Tisch R, McDevitt H (1996) Insulin-dependent diabetes mellitus. Cell 85: 291–297. 8616883 10.1016/s0092-8674(00)81106-x

[4] Kuniyasu Y, Takahashi T, Itoh M, Shimizu J, Toda G, et al (2000) Naturally anergic and suppressive CD25(+)CD4(+) T cells as a functionally and phenotypically distinct immunoregulatory T cell subpopulation. Int Immunol 12: 1145–1155. 10917889 10.1093/intimm/12.8.1145

[5] Todd JA, Walker NM, Cooper JD, Smyth DJ, Downes K, et al (2007) Robust associations of four new chromosome regions from genome-wide analyses of type 1 diabetes. Nat Genet 39: 857–864. 17554260 10.1038/ng2068 PMC2492393

[6] Lowe CE, Cooper JD, Brusko T, Walker NM, Smyth DJ, et al (2007) Large-scale genetic fine mapping and genotype-phenotype associations implicate polymorphism in the IL2RA region in type 1 diabetes. Nat Genet 39: 1074–1082. 17676041 10.1038/ng2102

[7] Vella A, Cooper JD, Lowe CE, Walker N, Nutland S, et al (2005) Localization of a type 1 diabetes locus in the IL2RA/CD25 region by use of tag single-nucleotide polymorphisms. Am J Hum Genet 76: 773–779. 15776395 10.1086/429843 PMC1199367

[8] Qu HQ, Montpetit A, Ge B, Hudson TJ, Polychronakos C (2007) Toward further mapping of the association between the IL2RA locus and type 1 diabetes. Diabetes 56: 1174–1176. 17395754 10.2337/db06-1555

[9] Hafler DA, Compston A, Sawcer S, Lander ES, Daly MJ, et al (2007) Risk alleles for multiple sclerosis identified by a genomewide study. N Engl J Med 357: 851–862. 17660530 10.1056/NEJMoa073493

[10] Stahl EA, Raychaudhuri S, Remmers EF, Xie G, Eyre S, et al (2010) Genome-wide association study meta-analysis identifies seven new rheumatoid arthritis risk loci. Nat Genet 42: 508–514. 20453842 10.1038/ng.582 PMC4243840

[11] Kim HP, Imbert J, Leonard WJ (2006) Both integrated and differential regulation of components of the IL-2/IL-2 receptor system. Cytokine Growth Factor Rev 17: 349–366. 16911870 10.1016/j.cytogfr.2006.07.003

[12] Lee GR, Kim ST, Spilianakis CG, Fields PE, Flavell RA (2006) T helper cell differentiation: regulation by cis elements and epigenetics. Immunity 24: 369–379. 16618596 10.1016/j.immuni.2006.03.007

[13] Lee PP, Fitzpatrick DR, Beard C, Jessup HK, Lehar S, et al (2001) A critical role for Dnmt1 and DNA methylation in T cell development, function, and survival. Immunity 15: 763–774. 11728338 10.1016/s1074-7613(01)00227-8

[14] Barrett JC, Clayton DG, Concannon P, Akolkar B, Cooper JD, et al (2009) Genome-wide association study and meta-analysis find that over 40 loci affect risk of type 1 diabetes. Nat Genet 41: 703–707. 19430480 10.1038/ng.381 PMC2889014

[15] Bradfield JP, Qu HQ, Wang K, Zhang H, Sleiman PM, et al (2011) A genome-wide meta-analysis of six type 1 diabetes cohorts identifies multiple associated loci. PLoS Genet 7: e1002293. 21980299 10.1371/journal.pgen.1002293 PMC3183083

[16] Fradin D, Le Fur S, Mille C, Naoui N, Groves C, et al (2012) Association of the CpG methylation pattern of the proximal insulin gene promoter with type 1 diabetes. PLoS One 7: e36278. 22567146 10.1371/journal.pone.0036278 PMC3342174

[17] Rakyan VK, Beyan H, Down TA, Hawa M, Maslau S, et al (2011) Identification of Type 1 Diabetes-Associated DNA Methylation Variable Positions That Precede Disease Diagnosis. PLoS Genet 7: e1002300. 21980303 10.1371/journal.pgen.1002300 PMC3183089

[18] Bell CG, Finer S, Lindgren CM, Wilson GA, Rakyan VK, et al (2010) Integrated genetic and epigenetic analysis identifies haplotype-specific methylation in the FTO type 2 diabetes and obesity susceptibility locus. PLoS One 5: e14040. 21124985 10.1371/journal.pone.0014040 PMC2987816

[19] Gibbs JR, van der Brug MP, Hernandez DG, Traynor BJ, Nalls MA, et al (2010) Abundant quantitative trait loci exist for DNA methylation and gene expression in human brain. PLoS Genet 6: e1000952. 20485568 10.1371/journal.pgen.1000952 PMC2869317

[20] Kerkel K, Spadola A, Yuan E, Kosek J, Jiang L, et al (2008) Genomic surveys by methylation-sensitive SNP analysis identify sequence-dependent allele-specific DNA methylation. Nat Genet 40: 904–908. 18568024 10.1038/ng.174

[21] Shoemaker R, Deng J, Wang W, Zhang K (2010) Allele-specific methylation is prevalent and is contributed by CpG-SNPs in the human genome. Genome Res 20: 883–889. 20418490 10.1101/gr.104695.109 PMC2892089

[22] Zhang D, Cheng L, Badner JA, Chen C, Chen Q, et al (2010) Genetic control of individual differences in gene-specific methylation in human brain. Am J Hum Genet 86: 411–419. 20215007 10.1016/j.ajhg.2010.02.005 PMC2833385

[23] Wellcome Trust Case Control Consortium (2007) Genome-wide association study of 14,000 cases of seven common diseases and 3,000 shared controls. Nature 447: 661–678. 17554300 10.1038/nature05911 PMC2719288

[24] Cho JH, Kimura H, Minami T, Ohgane J, Hattori N, et al (2001) DNA methylation regulates placental lactogen I gene expression. Endocrinology 142: 3389–3396. 11459782 10.1210/endo.142.8.8347

[25] Hattori N, Imao Y, Nishino K, Ohgane J, Yagi S, et al (2007) Epigenetic regulation of Nanog gene in embryonic stem and trophoblast stem cells. Genes Cells 12: 387–396. 17352742 10.1111/j.1365-2443.2007.01058.x

[26] Hattori N, Nishino K, Ko YG, Ohgane J, Tanaka S, et al (2004) Epigenetic control of mouse Oct-4 gene expression in embryonic stem cells and trophoblast stem cells. J Biol Chem 279: 17063–17069. 14761969 10.1074/jbc.M309002200

[27] Imamura T, Ohgane J, Ito S, Ogawa T, Hattori N, et al (2001) CpG island of rat sphingosine kinase-1 gene: tissue-dependent DNA methylation status and multiple alternative first exons. Genomics 76: 117–125. 11560121 10.1006/geno.2001.6607

[28] Nishino K, Hattori N, Tanaka S, Shiota K (2004) DNA methylation-mediated control of Sry gene expression in mouse gonadal development. J Biol Chem 279: 22306–22313. 14978045 10.1074/jbc.M309513200

[29] Shen S, Sandoval J, Swiss VA, Li J, Dupree J, et al (2008) Age-dependent epigenetic control of differentiation inhibitors is critical for remyelination efficiency. Nat Neurosci 11: 1024–1034. 19160500 10.1038/nn.2172 PMC2656679

[30] Yagi S, Hirabayashi K, Sato S, Li W, Takahashi Y, et al (2008) DNA methylation profile of tissue-dependent and differentially methylated regions (T-DMRs) in mouse promoter regions demonstrating tissue-specific gene expression. Genome Res 18: 1969–1978. 18971312 10.1101/gr.074070.107 PMC2593572

[31] Byun HM, Siegmund KD, Pan F, Weisenberger DJ, Kanel G, et al (2009) Epigenetic profiling of somatic tissues from human autopsy specimens identifies tissue- and individual-specific DNA methylation patterns. Hum Mol Genet 18: 4808–4817. 19776032 10.1093/hmg/ddp445 PMC4481584

[32] Cui H, Cruz-Correa M, Giardiello FM, Hutcheon DF, Kafonek DR, et al (2003) Loss of IGF2 imprinting: a potential marker of colorectal cancer risk. Science 299: 1753–1755. 12637750 10.1126/science.1080902

[33] Talens RP, Boomsma DI, Tobi EW, Kremer D, Jukema JW, et al (2010) Variation, patterns, and temporal stability of DNA methylation: considerations for epigenetic epidemiology. FASEB J 24: 3135–3144. 20385621 10.1096/fj.09-150490

[34] Furtado GC, Curotto de Lafaille MA, Kutchukhidze N, Lafaille JJ (2002) Interleukin 2 signaling is required for CD4(+) regulatory T cell function. J Exp Med 196: 851–857. 12235217 10.1084/jem.20020190 PMC2194060

[35] Sakaguchi S, Sakaguchi N, Asano M, Itoh M, Toda M (1995) Immunologic self-tolerance maintained by activated T cells expressing IL-2 receptor alpha-chains (CD25). Breakdown of a single mechanism of self-tolerance causes various autoimmune diseases. J Immunol 155: 1151–1164. 7636184

[36] Jung LK, Hara T, Fu SM (1984) Detection and functional studies of p60–65 (Tac antigen) on activated human B cells. J Exp Med 160: 1597–1602. 6092512 10.1084/jem.160.5.1597 PMC2187500

[37] Waldmann TA, Goldman CK, Robb RJ, Depper JM, Leonard WJ, et al (1984) Expression of interleukin 2 receptors on activated human B cells. J Exp Med 160: 1450–1466. 6092511 10.1084/jem.160.5.1450 PMC2187491

[38] Caligiuri MA, Murray C, Robertson MJ, Wang E, Cochran K, et al (1993) Selective modulation of human natural killer cells in vivo after prolonged infusion of low dose recombinant interleukin 2. J Clin Invest 91: 123–132. 7678599 10.1172/JCI116161 PMC330005

[39] Caligiuri MA, Zmuidzinas A, Manley TJ, Levine H, Smith KA, et al (1990) Functional consequences of interleukin 2 receptor expression on resting human lymphocytes. Identification of a novel natural killer cell subset with high affinity receptors. J Exp Med 171: 1509–1526. 1692080 10.1084/jem.171.5.1509 PMC2187895

[40] Fehniger TA, Cooper MA, Nuovo GJ, Cella M, Facchetti F, et al (2003) CD56bright natural killer cells are present in human lymph nodes and are activated by T cell-derived IL-2: a potential new link between adaptive and innate immunity. Blood 101: 3052–3057. 12480696 10.1182/blood-2002-09-2876

[41] Bosco MC, Espinoza-Delgado I, Rowe TK, Malabarba MG, Longo DL, et al (1997) Functional role for the myeloid differentiation antigen CD14 in the activation of human monocytes by IL-2. J Immunol 159: 2922–2931. 9300716

[42] Kniep EM, Strelow I, Lohmann-Matthes ML (1992) The monocyte interleukin-2 receptor light chain: production of cell-associated and soluble interleukin-2 receptor by monocytes. Immunology 75: 299–304. 1551692 PMC1384710

[43] Kronin V, Vremec D, Shortman K (1998) Does the IL-2 receptor alpha chain induced on dendritic cells have a biological function? Int Immunol 10: 237–240. 9533452 10.1093/intimm/10.2.237

[44] Velten FW, Rambow F, Metharom P, Goerdt S (2007) Enhanced T-cell activation and T-cell-dependent IL-2 production by CD83+, CD25high, CD43high human monocyte-derived dendritic cells. Mol Immunol 44: 1544–1550. 17023048 10.1016/j.molimm.2006.08.020

