# Supplementary figures and images for: CpG Methylation Changes within the IL2RA Promoter in Type 1 Diabetes of Childhood Onset
Source: PLoS One. 2013 Jul 12;8(7):e68093. doi: 10.1371/journal.pone.0068093 (PMC3709990; doi:10.1371/journal.pone.0068093)

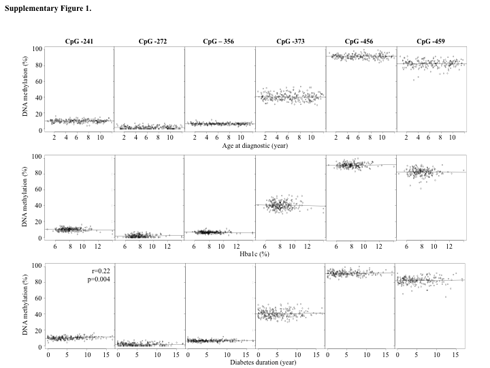

Supplement: Figure S1 — Lack of correlation between age at diagnostic, Hba1c and diabetes duration and IL2RA promoter methylation in T1D patients. Only CpG −241 showed a slight trend with diabetes duration (p = 0.004). (TIF) [file pone.0068093.s001.tif]

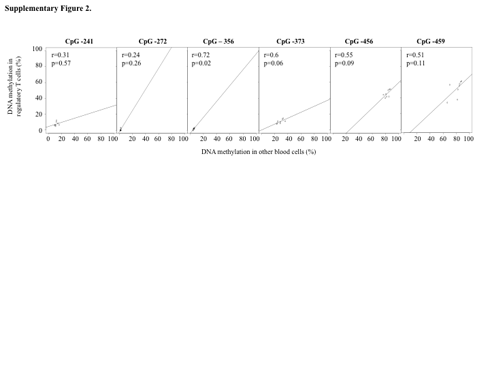

Supplement: Figure S2 — Correlation between IL2RA promoter methylation in regulatory T cells and other blood cells from 8 healthy individuals. Only CpG −356 showed a significant correlation however, all other CpG seemed correlated but failed to reach the significativity because of the weak number of participants. (TIF) [file pone.0068093.s002.tif]
