# Supplementary material for: CpG Methylation Changes within the IL2RA Promoter in Type 1 Diabetes of Childhood Onset
Source: PLoS One. 2013 Jul 12;8(7):e68093. doi: 10.1371/journal.pone.0068093 (PMC3709990; doi:10.1371/journal.pone.0068093)
Supplement: Table S1 — List of the 77 diabetic centers by alphabetic order participating to the ISIS-DIAB network. (DOCX) [file pone.0068093.s003.docx]

Supplemental Table 1. List of the 77 diabetic centers by alphabetic order participating to the ISIS-DIAB network.

| **Center** | **Principal Investigator** | **Center** | **Principal Investigator** |
| --- | --- | --- | --- |
| **ANGERS** | Pr Régis Coutant | **BOULOGNE BILLANCOURT** | Dr Chantal Stheneur |
| **ARRAS** | Dr Armelle Pambou | **CLAMART** | Dr Vincent Gajdos |
| **BETHUNE** | Dr Chantal Stuckens | **HYERES** | Dr Jamil Khoury |
| **BORDEAUX** | Pr Pascal Barat | **LILLE** | Dr Jean-Pierre Cappoen |
| **BOULOGNE SUR MER** | Dr Sylviane Fournier | **LIMOGES** | Dr Anne Lienhardt-Roussie |
| **BREST** | Dr Emmanuel Sonnet | **LYON** | Pr Marc Nicolino et Pr Pierre Chatelain |
| **BREST** | Dr Chantal Metz | **MONTFERMEIL** | Dr Philippe Talon |
| **CAMBRAI** | Dr Ramona Nicolescu | **NICE (Lenval)** | Dr Elisabeth Baechler |
| **CHOLET** | Dr Catherine Radet | **PARIS HOTEL DIEU** | Dr Etienne Larger |
| **DOUAI** | Dr Daniel Dervaux | **PARIS ST LOUIS** | Dr Jean-François Gautier |
| **DUNKERQUE** | Dr Guy-André Loeuille | **SAINTES** | Dr Catherine Boniface |
| **LA ROCHE SUR YON** | Dr Jean-Pierre Brossier | **TOULON** | Dr Berthe Razafimahefa |
| **LAVAL** | Dr Joachim Bassil | **AVIGNON** | Dr Florence Joubert |
| **LE HAVRE** | Dr Bernard Leluyer | **COMPIEGNE** | Dr Christine Vervel |
| **LE MANS** | Dr Françoise Labay | **PARIS LARIBOISIERE** | Dr Marie Laloi-Michelin |
| **LENS** | Dr Isabelle Guémas | **PAU** | Dr Bénédicte Guérin |
| **LILLE** | Dr Jacques Weill | **ARMENTIERES** | Dr Sophie Caudrelier |
| **MARSEILLE (La Timone)** | Dr Rachel Reynaud | **VALENCIENNES** | Dr Odile Verier-Mine |
| **MAUBEUGE** | Dr Agnès Tamboura | **AIX EN PROVENCE** | Dr Dominique Thevenieau |
| **MERIGNAC** | Dr Hervé Dubourg | **ANGERS** | Dr Pierre-Henri Ducluzeau |
| **MONT-DE-MARSAN** | Dr Marie-Laure Pignol | **ANGOULEME** | Dr Gérard Pointecouteau |
| **NANCY** | Dr Stéphanie Jellimann | **AVIGNON** | Dr Eric Benamo |
| **NANTES** | Dr Lucy Chaillous | **BAR LE DUC** | Dr Philippe Jan |
| **NANTES** | Dr Sabine Baron | **BELFORT-MONTBELIARD** | Dr Estelle Marcoux |
| **NICE** | Dr Marie-Noëlle Bortoluzzi | **BRIVE** | Dr Anne Fargeot-Espaliat |
| **PARIS BICETRE** | Pr Pierre Bougnères | **CAEN** | Pr Yves Reznik |
| **PONTOISE** | Dr Laetitia Pantalone | **CLERMONT FERRAND** | Dr Daniel Terral |
| **RENNES** | Dr Marc De Kerdanet | **DIJON** | Dr Candace Ben Signor |
| **ROUEN** | Dr Marie-Claire Wieliczko | **LIMOGES** | Dr Sylvie Nadalon |
| **SAINT-AVOLD** | Dr François Kurtz | **MARSEILLE (St Joseph)** | Dr Jacques Cohen |
| **SAINT-LO** | Dr Norbert Laisney | **MARSEILLE CHU NORD** | Dr Catherine Atlan |
| **SAINT-NAZAIRE** | Dr Guilhem Parlier | **NIORT** | Dr Patrick Flamen |
| **TOULOUSE** | Pr Pierre Gourdy | **REIMS** | Pr Brigitte Delemer |
| **TOURS** | Dr Myriam Pépin-Donat | **RENNES** | Dr Isabelle Guilhem |
| **VALENCIENNES** | Dr Anne Gourdin | **SAINT ETIENNE** | Dr Odile Richard |
| **VILLEFRANCHE SUR SAONE** | Dr Michèle Chambon | **THIONVILLE** | Dr Mirela Codreanu |
| **AMIENS** | Dr Rachel Desailloud | **TOURS** | Pr Pierre Lecomte |
| **AMIENS** | Dr Hélène Bony-Trifunovic | **VALENCE** | Dr Marina Raoulx |
| **BESANCON** | Dr Brigitte Mignot et Anne-Marie Bertrand | **VIENNE** | Dr Catherine Dumont |
